# Sodium butyrate ameliorates gut dysfunction and motor deficits in a mouse model of Parkinson’s disease by regulating gut microbiota

**DOI:** 10.3389/fnagi.2023.1099018

**Published:** 2023-01-25

**Authors:** Yi Zhang, Shaoqing Xu, Yiwei Qian, Chengjun Mo, Penghui Ai, Xiaodong Yang, Qin Xiao

**Affiliations:** ^1^Department of Neurology and Institute of Neurology, Ruijin Hospital, Shanghai Jiao Tong University School of Medicine, Shanghai, China; ^2^Department of Geriatrics, Ruijin Hospital, Shanghai Jiao Tong University School of Medicine, Shanghai, China

**Keywords:** sodium butyrate, gut microbiota, Parkinson’s disease, short chain fatty acids, glucagon like peptide-1

## Abstract

**Background:**

A growing body of evidence showed that gut microbiota dysbiosis might be associated with the pathogenesis of Parkinson’s disease (PD). Microbiota-targeted interventions could play a protective role in PD by regulating the gut microbiota-gut-brain axis. Sodium butyrate (NaB) could improve gut microbiota dysbiosis in PD and other neuropsychiatric disorders. However, the potential mechanism associated with the complex interaction between NaB and gut microbiota-gut-brain communication in PD needs further investigation.

**Methods:**

C57BL/6 mice were subjected to a rotenone-induced PD model and were treated intragastrically with NaB for 4 weeks. The gut function and motor function were evaluated. The α-synuclein expression in colon and substantia nigra were detected by western blotting. Tyrosine hydroxylase (TH)-positive neurons in substantia nigra were measured by immunofluorescence. Moreover, gut microbiota composition was analyzed by 16S rRNA sequencing. Fecal short chain fatty acids (SCFAs) levels were determined by liquid chromatography tandem mass spectrometry (LC–MS). The levels of glucagon like peptide-1 (GLP-1) in tissues and serum were evaluated using enzyme-linked immunosorbent assay (ELISA).

**Results:**

NaB ameliorated gut dysfunction and motor deficits in rotenone-induced mice. Meanwhile, NaB protected against rotenone-induced α-synuclein expression in colon and substantia nigra, and prevented the loss of TH-positive neurons. In addition, NaB could remodel gut microbiota composition, and regulate gut SCFAs metabolism, and restore GLP-1 levels in colon, serum, and substantia nigra in PD mice.

**Conclusion:**

NaB could ameliorate gut dysfunction and motor deficits in rotenone-induced PD mice, and the mechanism might be associated with the regulation of gut microbiota dysbiosis.

## Introduction

Parkinson’s disease (PD) is the second most common neurodegenerative disease, characterized by the selective loss of dopaminergic neurons and the formation of lewy bodies comprised mainly of α-synuclein (Armstrong and Okun, 2020). With the progressive loss of dopaminergic neurons, the synthesis of dopamine in substantia nigra and striatum is decreased, resulting in resting tremor, rigidity, and bradykinesia (Cacabelos, 2017). In addition to the well-known motor symptoms observed in PD, early non-motor deficits, for example gastrointestinal symptoms, have also been given considerable attention (Lanza et al., 2022). The gastrointestinal symptoms seen in patients with PD include drooling, dysphagia, malnutrition, impaired gastric emptying, and constipation (Fasano et al., 2015). Constipation is one of the most prevalent non-motor symptoms in PD and may predate the diagnosis of PD by over a decade (Adams-Carr et al., 2016; Knudsen et al., 2017). In addition, previous study found that α-synuclein pathology was observed in colon tissues obtained 2–5 years prior to the onset of PD (Shannon et al., 2012). In rotenone-induced PD mice, α-synuclein was also detected in the colon earlier than brain (Yang et al., 2017). Thus, it has been hypothesized that the pathology of PD may originate from gut (Braak et al., 2003; Scheperjans et al., 2015).

Gut contains trillions of bacteria, which is emerging as key players in governing the host health and disease (Heintz-Buschart and Wilmes, 2018). The gut microbiota attracts more and more attention in the pathogenesis of neurodegenerative diseases (Roy Sarkar and Banerjee, 2019; Gubert et al., 2020). The dysbiosis of gut microbiota has been widely recognized in patients with PD (Nishiwaki et al., 2020; Romano et al., 2021). Sampson et al. transplanted the feces from patients with PD into α-synuclein overexpressing mice, which significantly aggravated motor deficits (Sampson, 2016), suggesting that the alteration of gut microbiota composition represented a risk factor for PD. Notably, the gut microbiota-targeted interventions, such as fecal microbiota transplantation and probiotics, could significantly promote the survival of dopaminergic neurons, increase the level of dopamine in striatum, and improve motor deficits through restoration of gut microbiota dysbiosis (Sun et al., 2018, 2021). Thus, approaches aiming to regulate the gut microbiota could be a potential strategy for the treatment of PD. Previous studies reported that sodium butyrate (NaB) could ameliorate gut microbiota dysbiosis and exert beneficial effects in neuropsychiatric disorders (Chen et al., 2019; Wang et al., 2021; Lanza et al., 2022; Yong et al., 2022). Avagliano et al. found that NaB could re-shape microbiota composition in a novel dual-hit model of PD (Avagliano et al., 2022). However, the potential mechanism associated with the complex interaction between NaB and gut microbiota-gut-brain communication in PD needs further investigation.

Gut microbiota can ferment dietary components to produce a variety of metabolites, such as short chain fatty acids (SCFAs), bile acids, and amino acids (Koutzoumis et al., 2020). SCFAs, mainly including acetic acid, propionic acid, butyric acid, isobutyric acid, valeric acid, and isovaleric acid, are the main end products of non-digestible carbohydrates fermented by gut microbiota (Cummings, 1983). Meanwhile, SCFAs may serve as the key mediators in the gut microbiota-gut-brain communication, especially inhibiting neuroinflammation and alleviating neurological damage (Dalile et al., 2019). The disturbance of SCFAs metabolism is observed in PD (Chen et al., 2022), which may be associated with gut microbiota dysbiosis. A growing body of evidence suggested that SCFAs might play a potential role in the pathogenesis of PD (Dalile et al., 2019; Aho et al., 2021; Mirzaei et al., 2021). Although the mechanism of SCFAs-mediated microbiota-gut-brain communication has not been fully elucidated in PD, glucagon like peptide-1 (GLP-1) may serve as one of the key regulatory factors (Dalile et al., 2019). GLP-1 is an insulin stimulating hormone secreted by intestinal endocrine L cells, and depends on GLP-1 receptor (GLP-1R) to transmit signals, participating in the regulation of blood glucose homeostasis (Holst, 2007). In recent years, the neuroprotective role of GLP-1 in central nervous system has attracted more and more attention (Salcedo et al., 2012). Enhancement of GLP-1 secretion by GLP-1R agonist can exert protective effects in PD (Kim et al., 2009; Athauda et al., 2017). However, whether GLP-1 is involved in the interaction between NaB and gut microbiota-gut-brain communication in PD has not been investigated.

In this study, we aimed to investigate the role of NaB in rotenone-treated PD mouse model, and try to explore the potential mechanisms involved.

## Materials and methods

### Animals and treatment

Male C57BL/6 mice (8–10 weeks, 20–25 g) were provided by the Shanghai Biomodel Organism Science & Technology Development Co., Ltd. The animals were housed (three per cage) in a specific pathogen free animal facility (ambient temperature: 20 ± 2°C; humidity: 50–65%) under a 12 h light/dark cycles, with free access to water and food. All experimental protocols were authorized by Animal Care Committee of Shanghai Jiao Tong University School of Medicine. Mice were randomly divided into four groups (*n* = 10 per group): control group; NaB group; rotenone group; rotenone + NaB group. Mice were treated by gavage with vehicle solution, NaB (200 mg/Kg), or rotenone (30 mg/Kg) once daily for 4 weeks, as previously described (Liu et al., 2017; Yang et al., 2017).

### Measurement of colon motility and behavioral test

One-hour stool frequency was measured to evaluate the colon motility (Greene et al., 2009). The measurements were performed between 9:00 and 11:00 on each day. Briefly, the stools within 1 h were collected, and stool water content was calculated from the difference between wet and dry weights. The results were normalized to body weight (Zhi et al., 2006). Motor deficits in rotenone-induced PD mouse model was evaluated using the open field test to measure the spontaneous locomotor activity (Walsh and Cummins, 1976), and pole test to measure the total time required to climb down the pole (Ogawa et al., 1985).

### Western blotting

The western blotting was performed as previously described (Zhang Y. et al., 2022). The colon and middle brain substantia nigra were rapidly dissected and lysed in RIPA lysis buffer (Cat.No. P0013B, Beyotime Institute of Biotechnology, Shanghai, China) containing protease and phosphatase inhibitor cocktails (Cat.No. P1045, Beyotime Institute of Biotechnology, Shanghai, China) and 1 mM phenyl-methylsulphonyl fluoride (PMSF; Cat.No. ST505, Beyotime Institute of Biotechnology, Shanghai, China). Tissue extracts were centrifuged at 14,000 g for 20 min at 4°C. Protein concentration was determined using the bicinchoninic acid (BCA) protein assay kit (Cat.No. P0012S, Beyotime Institute of Biotechnology, Shanghai, China). The samples (30 μg) were separated using 12% sodium dodecyl sulfate–polyacrylamide gel electrophoresis (SDS-PAGE) and transferred to immobilon polyvinylidene difluoride (PVDF) membrane. The membranes were probed overnight at 4°C for the expression of α-synuclein (1:1000, Cat.No. 2628S, Cell Signaling Technology, Beverly, MA, United States) and phospho-Ser129 α-synuclein (1:1000, Cat.No. 23706, Cell Signaling Technology, Beverly, MA, United States). Then, the membranes were incubated with the appropriate horseradish-peroxidase (HRP)-conjugated secondary antibodies (1:10000, Cat.No. 7074, Cell Signaling Technology, Beverly, MA, United States) at room temperature for 2 h, and protein bands were visualized using the enhanced chemiluminescence detection system.

### Immunofluorescence

After the mice were sacrificed, the colon and brain were removed and fixed with 4% paraformaldehyde solution. These samples were embedded in paraffin after dehydration and cut into 5 μm sections. The sections were probed for βIII-tubulin, phospho-Ser129 α-synuclein, and tyrosine hydroxylase (TH) using immunochemistry staining. The slides were incubated at 4°C overnight with primary antibody. The sections were then incubated with secondary antibody. The slides were observed under light microscopy and photographed.

### Fecal sample collection, DNA extraction, amplification and sequencing of 16S rRNA gene

Fecal samples were collected immediately after expulsion. Fecal microbial genomic DNA was extracted using QIAamp DNA stool mini kit (Qiagen, Hilden, Germany) following the manufacturer’s instructions. The V3-V4 regions of 16S rRNA genes were amplified by PCR using the following primers: 338F 5’-ACTCCTACGGGAGGCAGCAG-3′ and 806R 5’-GGACTACHVGGGTWTCTAAT-3′. PCR reactions were performed in 30 μl mixtures containing 15 μl of 2 × KAPA Library Amplification ReadyMix, 1 μl of each primer (10 μM), and 50 ng of template DNA and ddH2O under the following cycling conditions: 95°C for 3 min, followed by 30 cycles at 98°C for 20 s, 58°C for 15 s, and 72°C for 20 s, then a final extension at 72°C for 5 min. After purification using AxyPrep DNA Gel Extraction Kit (Axygen Biosciences, Union City, CA, United States), the library preparation was performed with NEXTFLEX Rapid DNA-Seq Kit (Bioo Scientific, Austin, Texas, United States). The pooled products were paired-end sequenced on the Illumina MiSeq platform (Illumina, San Diego, CA, United States) according to standard protocols.

### Microbiota analysis

Raw sequencing data were demultiplexed and quality-filtered, and chimeric sequences were removed using the Divisive Amplicon Denoising Algorithm 2 (DADA2; Callahan et al., 2016) with the open-source software Quantitative Insights Into Microbial Ecology 2 (QIIME2). Taxonomical assignment was performed using the SILVA database (Quast et al., 2013). The amplicon sequence variants were used for alpha diversity indices, including Ace, Chao, Shannon, and Simpson. For beta-diversity, the dissimilarities (distances) were calculated and used for ordination by principal coordinates analysis (PCoA), which was performed for all dimension reduction analyses. Significant differences were assessed using permutational multivariate analysis of variance (PERMANOVA) with 999 permutations.

### Liquid chromatography–tandem mass spectrometry (LC–MS) analysis for fecal SCFAs

The freeze-dried fecal samples were thawed in an ice-bath, and about 5 mg of each sample was weighted. Then, 20 μl ultrapure water was added, and 120 μl methanol containing internal standards solution was used to extract the metabolites. The samples were homogenated for 3 min and centrifuged at 13500 g for 10 min. Then, 30 μl supernatant was transferred to a 96-well plate for derivatization using the Biomek 4,000 workstation (Biomek 4,000; Beckman Coulter, Inc., Brea, CA, United States). After derivatization, 400 μl ice-cold 50% methanol solution was added to dilute the samples. Then, the plate was stored at-20°C for 20 min and centrifugated at 4000 g for 30 min. Next, 135 μl of supernatant was transferred to a new 96-well plate for LC–MS analysis. LC–MS analyses were performed using an ultra-performance liquid chromatography coupled to tandem mass spectrometry (UPLC–MS) system (ACQUITY UPLC-Xevo TQ-S; Waters Corp., Milford, MA, United States) as previously described (Xie et al., 2021). The raw data files generated by LC–MS were processed using the MassLynx software (version 4.1; Waters, Milford, MA, USA) to perform peak integration, calibration, and quantitation for each metabolite.

### Measurement of GLP-1 in serum, colon, and substantia nigra using enzyme-linked immunosorbent assay

Blood samples from retro-orbital venous plexus were obtained. The samples were centrifuged with 1,000 g for 20 min to collect the serum. The homogenates from colon and middle brain substantia nigra were prepared. The levels of GLP-1 in colon, serum, and substantia nigra were measured using ELISA kit according to the manufacturer’s instructions. The GLP-1 levels in the tissue samples were normalized to protein concentrations.

### Statistical analysis

All data were graphed, and statistical analyses were performed using GraphPad Prism 5 software (GraphPad Software, San Diego, CA, United States), SPSS software (version 22.0, IBM Corporation, Armonk, NY, United States), and R software (version 4.0.3; R Foundation for Statistical Computing, Vienna, Austria). Statistical analysis was conducted using the Student’s t-test or Mann–Whitney U test for comparisons of two factors, or using one-way ANOVA followed by Tukey’s *post-hoc* tests for comparisons of multiple factors. All bar graphs show mean ± standard error of mean (SEM). A *p* value of less than 0.05 was considered significant.

## Results

### NaB alleviates rotenone-induced gut dysfunction and motor deficits

**Figure 1:**
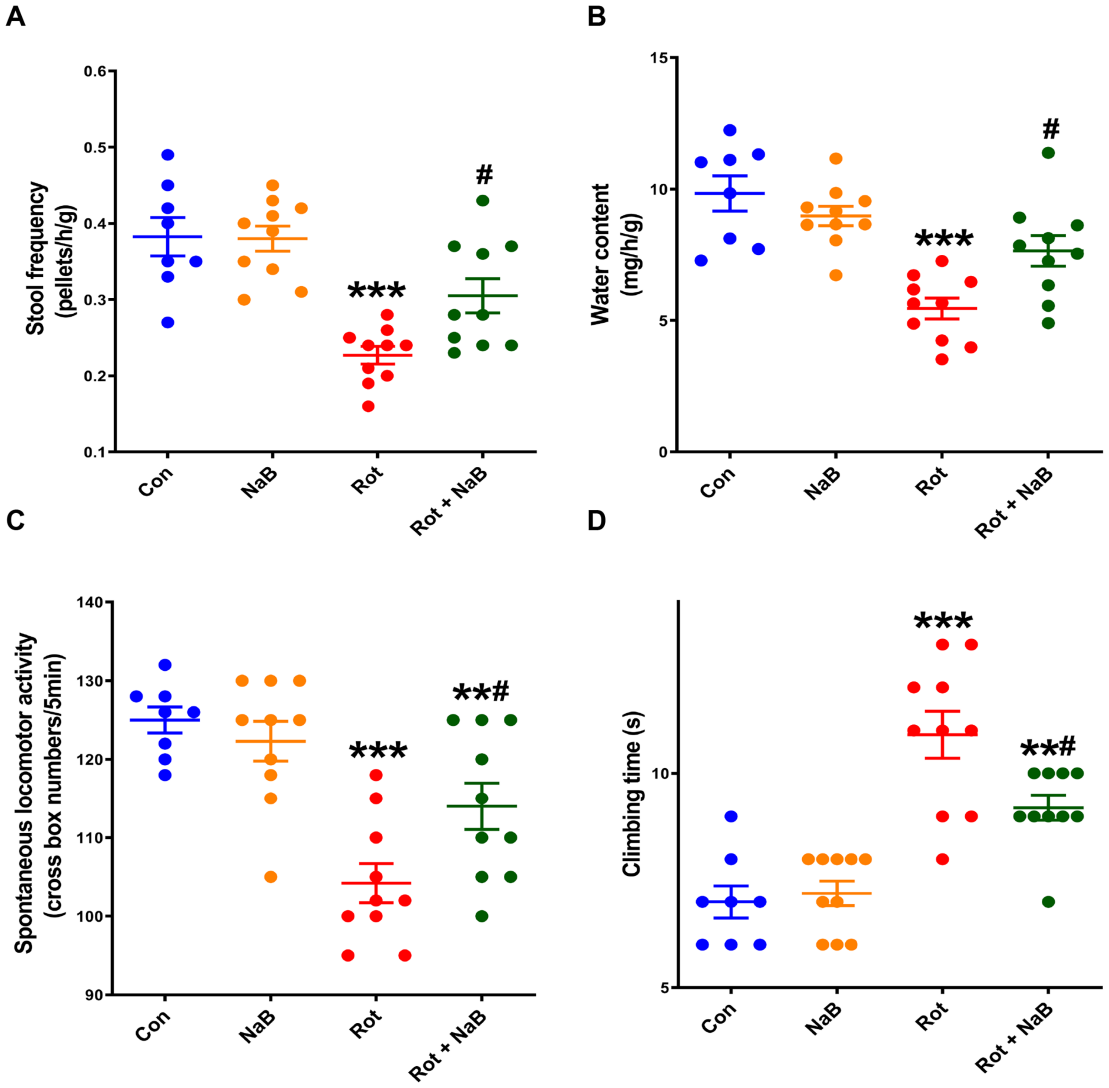
NaB alleviates rotenone-induced gut dysfunction and motor deficits. **(A)** Stool frequency and **(B)** water content evaluation for gut function. **(C)** Spontaneous locomotor activity and **(D)** climbing time measurements for motor function. *n* = 8–10 per group. The data represent the mean ± SEM. **p < 0.01, ***p < 0.001 vs. Con group; #*p* < 0.05 vs. Rot group. Con: control; NaB: sodium butyrate; Rot: rotenone.

We first investigated the potential effect of NaB on gut function in rotenone-induced PD mice. The stool frequency and water content were lower in rotenone-induced PD mice compared with the control group. NaB treatment alleviated rotenone-induced reduction of stool frequency and water content (Figures 1A,B).

Then we investigated the effect of NaB on motor function in rotenone-induced PD mice. Behavioral tests showed reduction in spontaneous locomotor activity and prolongation in climbing time in rotenone-induced PD mice compared with the control group. NaB treatment improved rotenone-induced reduction of spontaneous locomotor activity and prolongation of climbing time (Figures 1C,D).

### NaB alleviates rotenone-induced α-synuclein pathology

**Figure 2:**
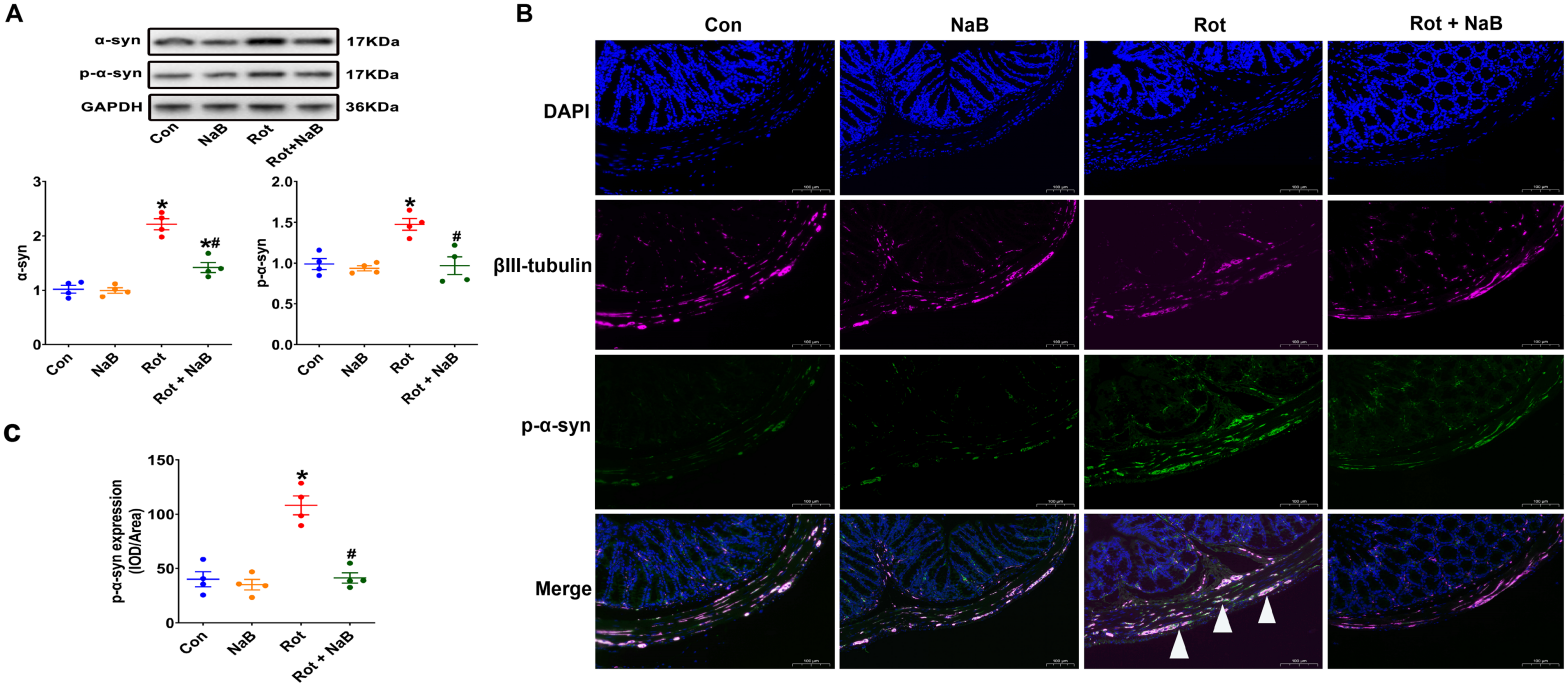
NaB alleviates rotenone-induced colonic α-synuclein pathology. **(A)** Representative blots of colonic α-syn and p-α-syn expression. **(B)** Representative photomicrographs of colon sections immunostained for DAPI, βIII-tubulin, and p-α-syn. The βIII-tubulin was used as the marker of colonic neurons. The presence of p-α-syn staining in colonic neurons was shown by the arrows. Scale bar = 100 μm. **(C)** Quantitative analysis of the mean fluorescence intensity of p-α-syn. *n* = 4 per group. The data represent the mean ± SEM. *p < 0.05 vs. Con group; #*p* < 0.05 vs. Rot group. α-syn: α-synuclein; Con: control; IOD: integrated optical density; NaB: sodium butyrate; p-α-syn: phospho-Ser129 α-synuclein; Rot: rotenone.

Because gut dysfunction is partly associated to the α-synuclein pathology in colon (Fasano et al., 2015), we then detected the expression of α-synuclein and phospho-Ser129 α-synuclein in colon tissue. The expression of α-synuclein and phospho-Ser129 α-synuclein in colon was increased in rotenone group compared with the control group. NaB treatment ameliorated rotenone-induced expression of colonic α-synuclein and phospho-Ser129 α-synuclein (Figure 2A). In addition, the phospho-Ser129 α-synuclein was mainly localized to the enteric neurons (Figure 2B). Rotenone increased the expression of phospho-Ser129 α-synuclein compared with the control group, while NaB treatment ameliorated rotenone-induced expression of phospho-Ser129 α-synuclein (Figures 2B,C).

**Figure 3:**
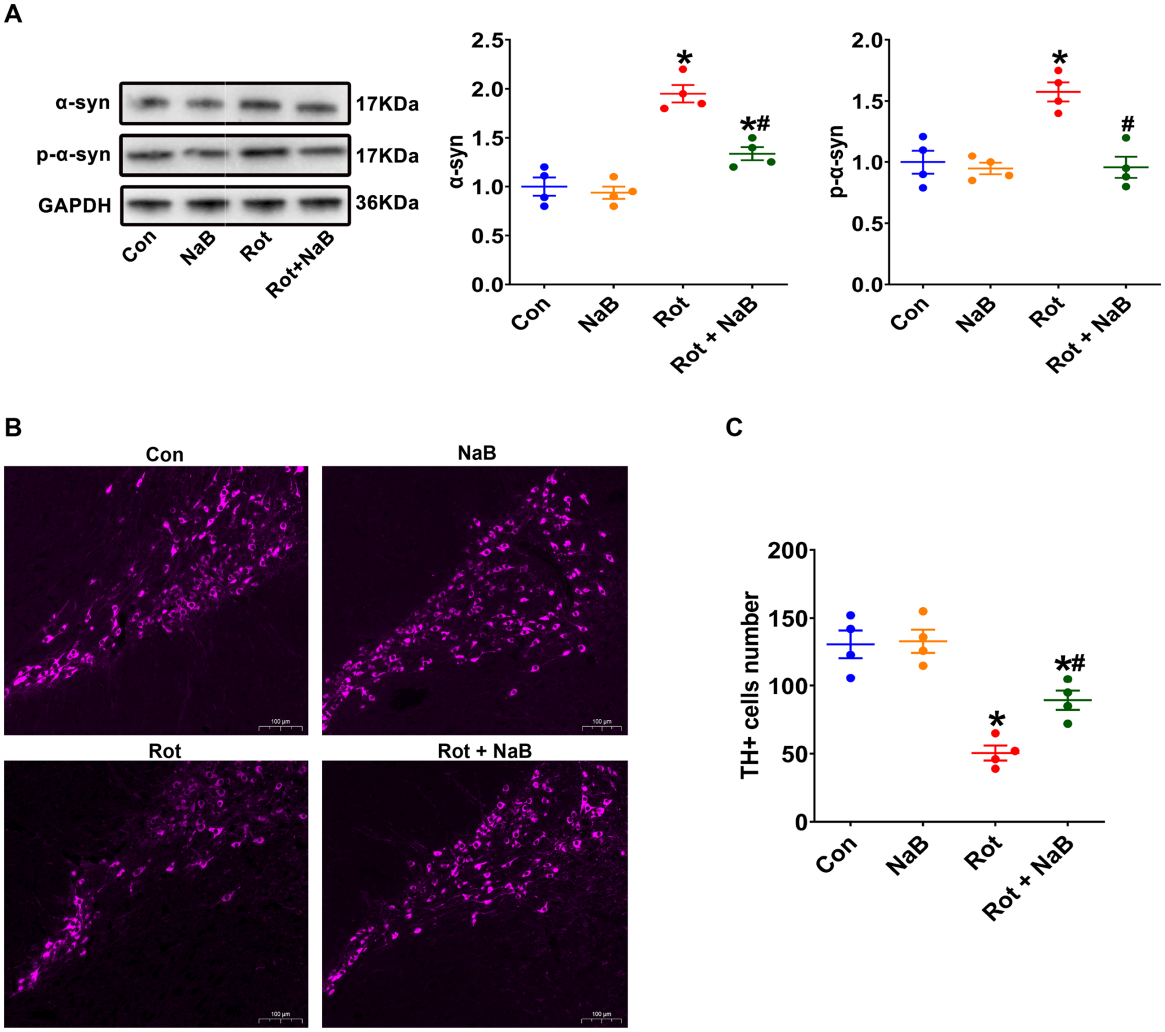
NaB alleviates rotenone-induced substantia nigra α-synuclein pathology. **(A)** Representative blots of α-syn and p-α-syn expression in substantia nigra. **(B)** Representative photomicrographs of substantia nigra sections immunostained for TH. Scale bar = 100 μm. **(C)** Quantitative analysis of the TH positive cells numbers. *n* = 4 per group. The data represent the mean ± SEM. *p < 0.05 vs. Con group; #*p* < 0.05 vs. Rot group. α-syn: α-synuclein; Con: control; NaB: sodium butyrate; p-α-syn: phospho-Ser129 α-synuclein; Rot: rotenone; TH: tyrosine hydroxylase.

We further observed the expression of α-synuclein and phospho-Ser129 α-synuclein in substantia nigra. The expression of α-synuclein and phospho-Ser129 α-synuclein were increased in rotenone group compared with the control group, which were ameliorated with NaB treatment (Figure 3A). Moreover, the numbers of TH-positive dopaminergic neurons were decreased in rotenone group compared with the control group, while NaB treatment could protect against the rotenone-induced loss of TH-positive dopaminergic neurons (Figures 3B,C).

### NaB improves rotenone-induced gut microbiota dysbiosis

**Figure 4:**
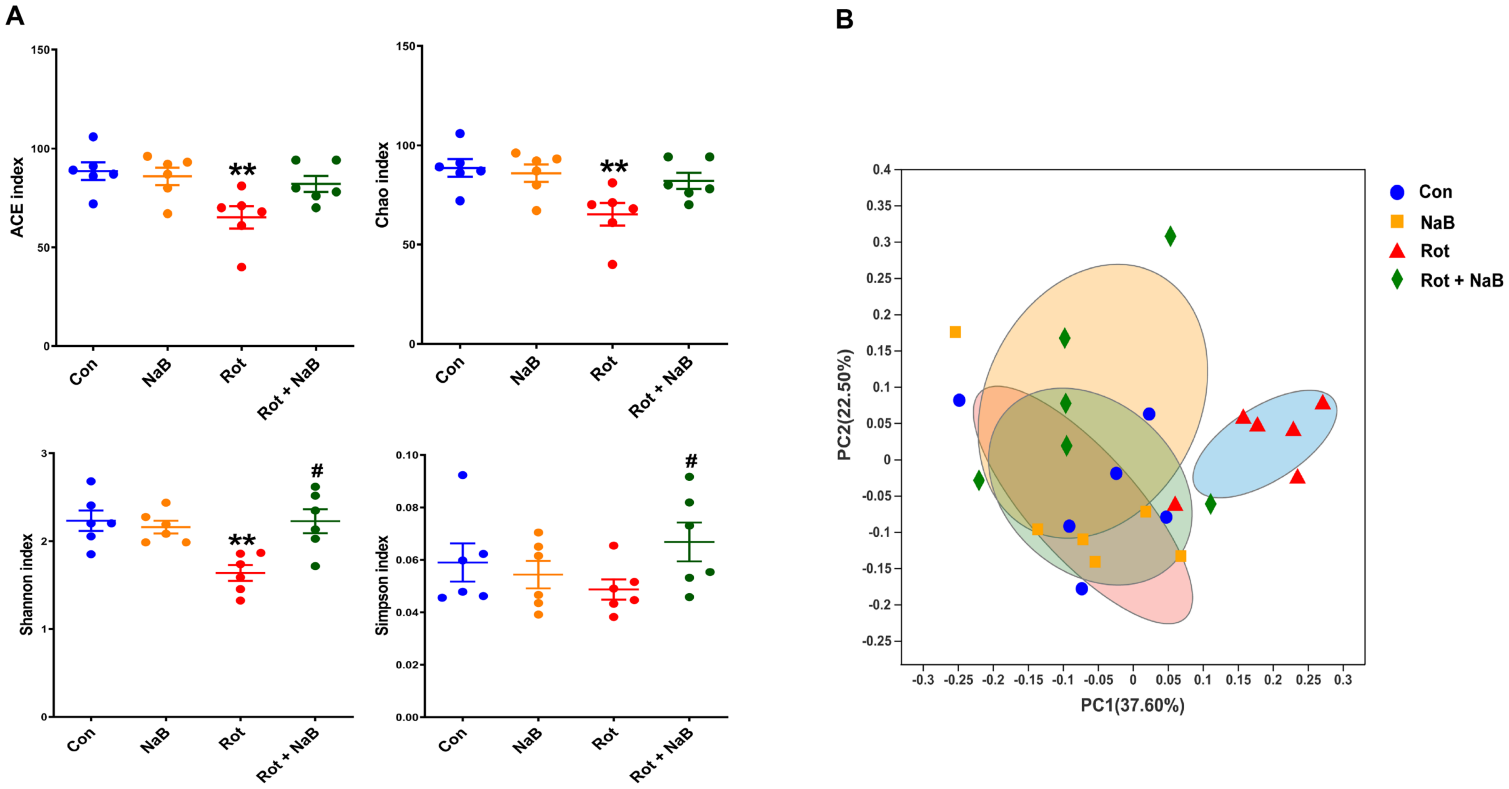
NaB reverses rotenone-induced alteration of gut microbial alpha and beta diversity. **(A)** Bar plots of alpha diversity indices, including ACE, Chao, Shannon, and Simpson index. **(B)** PCoA plots of the beta-diversity with Bray-Curtis measures. *n* = 6 per group. The data represent the mean ± SEM. **p < 0.01 vs. Con group; #*p* < 0.05 vs. Rot group. Con: control; NaB: sodium butyrate; PCoA: principal coordinate analysis; Rot: rotenone.

We performed 16S rRNA sequencing to investigate the possible role of NaB on gut microbiota, which has been reported to be regulated by NaB (Chen et al., 2019; Wang et al., 2021; Lanza et al., 2022; Yong et al., 2022). We performed gut microbial analysis based on alpha and beta diversity measures, which provided a holistic view of the gut microbiota composition. The alpha diversity indices of ACE, Chao, and Shannon were lower in rotenone-induced PD mice compared with the control group, while NaB treatment reversed the reduction of Shannon index (Figure 4A). The beta diversity refers to the extent of similarity among microbial communities, which was evaluated with PCoA based on Bray-Curtis. Notably, rotenone yielded a significant difference of beta diversity compared with the control group, while NaB treatment restored the rotenone-induced alteration of beta diversity (Figure 4B).

**Figure 5:**
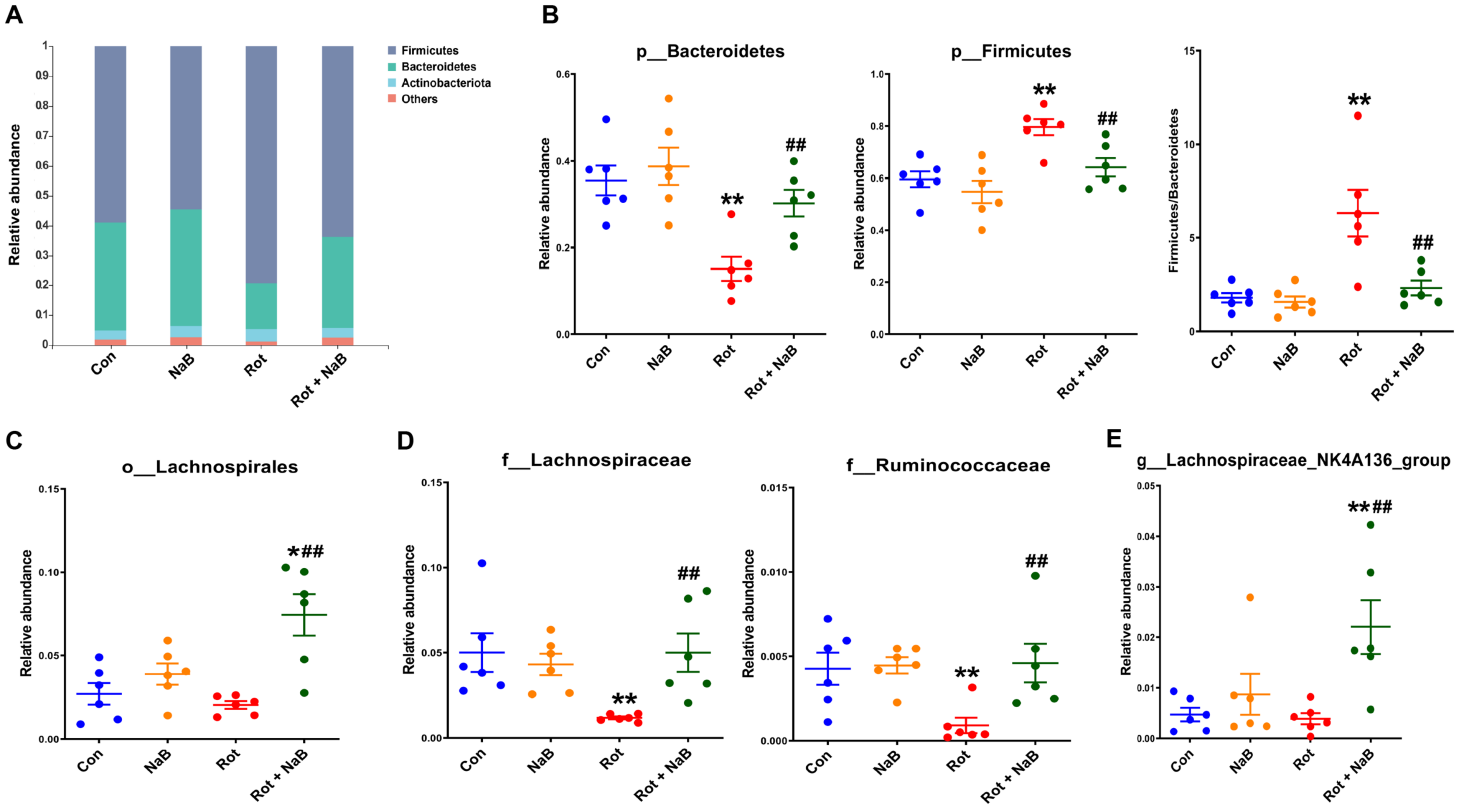
NaB reverses rotenone-induced alteration of gut microbial composition. **(A)** Bar plots of relative abundance of different groups at phylum level. Bacterial taxa with different abundance between groups at **(B)** phylum, **(C)** order, **(D)** family, and **(E)** genus level. *n* = 6 per group. The data represent the mean ± SEM. *p < 0.05, **p < 0.01 vs. Con group; ##*p* < 0.01 vs. Rot group. Con: control; NaB: sodium butyrate; Rot: rotenone.

To investigate the potential microbial taxa contributing to the gut microbiota dysbiosis, we identified and compared the bacterial abundance among different groups. At the phyla level, Firmicutes and Bacteroidetes represented the most abundant bacterial taxa (Figure 5A). The relative abundance of Firmicutes was higher, and the relative abundance of Bacteroidetes was lower in rotenone-induced mice compared with the control group, while NaB treatment protected against the higher abundance of Firmicutes and lower abundance of Bacteroidetes. In addition, the ratio of Firmicutes/Bacteroidetes was higher in rotenone group compared with the control group, while NaB treatment reversed the increased ratio of Firmicutes/Bacteroidetes (Figure 5B). At the order level, NaB treatment increased the relative abundance of Lachnospirales compared with rotenone-induced mice (Figure 5C). At the family level, the relative abundance of Lachnospiraceae and Ruminococcaceae were lower in rotenone-induced mice compared with the control group, while NaB treatment restored the reduction of the relative abundance of Lachnospiraceae and Ruminococcaceae in rotenone-induced mice (Figure 5D). At the genus level, NaB treatment improved the relative abundance of Lachnospiraceae_NK4A136_group in rotenone-induced mice (Figure 5E).

### NaB improves rotenone-induced fecal SCFAs metabolism disturbance

**Figure 6:**
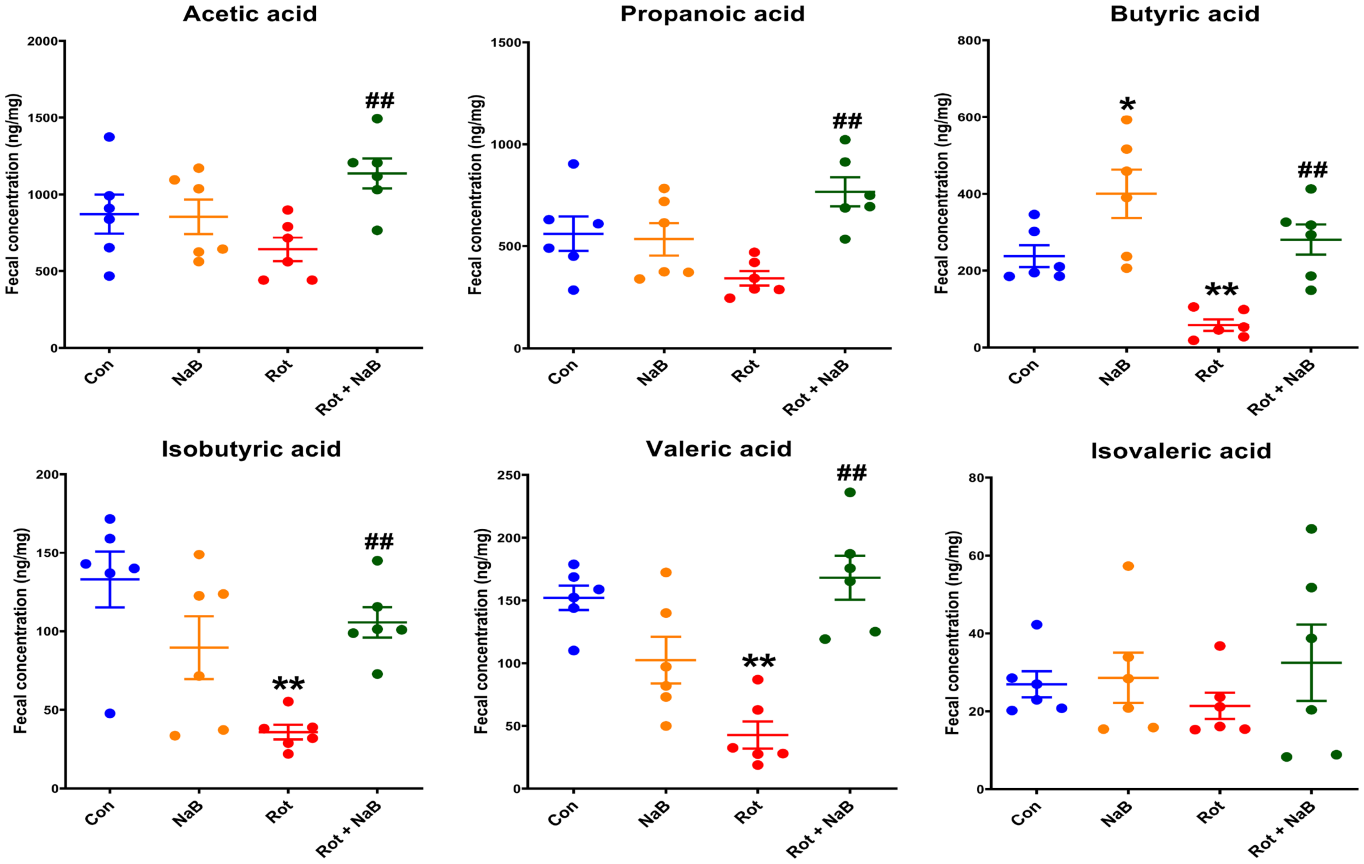
NaB improves rotenone-induced fecal SCFAs metabolism disturbance. Bar plots of the fecal acetate, propionate, butyric acid, isobutyric acid, valeric acid, and isovaleric acid levels. *n* = 6 per group. The data represent the mean ± SEM. *p < 0.05, **p < 0.01 vs. Con group; ##*p* < 0.01 vs. Rot group. Con: control; NaB: sodium butyrate; Rot: rotenone; SCFAs: short chain fatty acids.

Since SCFAs may serve as the potential factors mediating the gut microbiota-gut-brain communication (Dalile et al., 2019), we measured the profiles of fecal SCFAs in different groups. Among the fecal SCFAs (acetic acid, propionic acid, butyric acid, isobutyric acid, valeric acid, and isovaleric acid) detected using LC–MS, fecal butyric acid, isobutyric acid, and valeric acid levels were decreased in rotenone-induced mice compared with the control group, which was restored with NaB treatment. In addition, NaB treatment improved the fecal acetic acid and propionic acid levels in rotenone-induced mice, although the levels of acetic acid and propionic acid were not influenced by rotenone treatment. NaB treatment alone increased the fecal butyric acid levels compared with control group (Figure 6).

### NaB improves rotenone-induced reduction of GLP-1 levels

**Figure 7:**
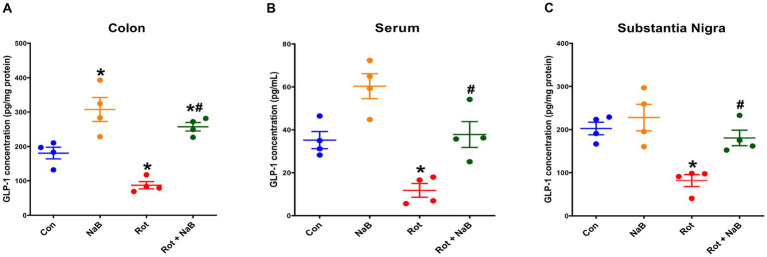
NaB improved rotenone-induced reduction of GLP-1 levels. Bar plots of GLP-1 levels in **(A)** colon, **(B)** serum, and **(C)** substantia nigra. *n* = 4 per group. The data represent the mean ± SEM. *p < 0.05 vs. Con group; #*p* < 0.05 vs. Rot group. Con: control; GLP-1: glucagon-like peptide-1; NaB: sodium butyrate; Rot: rotenone.

To further explore whether GLP-1 was involved in the mechanism underlying the protective role of NaB, we investigated the effect of NaB on GLP-1 levels in colon, serum, and substantia nigra. The GLP-1 levels in colon (Figure 7A), serum (Figure 7B), and substantia nigra (Figure 7C) were lower in rotenone-induced mice than that in control group. NaB treatment ameliorated the reduction of GLP-1 levels in colon, serum, and substantia nigra in rotenone-induced mice. NaB treatment alone increased the GLP-1 levels in colon, other than serum or substantia nigra.

## Discussion

The gut microbiota dysbiosis may be related to the pathogenesis of PD, and gut microbiota remodeling could exert beneficial effects (Liu et al., 2020; Lorente-picón and Laguna, 2021). NaB has been reported to regulate the gut microbiota composition in PD mouse model (Avagliano et al., 2022) and other neuropsychiatric disorders (Chen et al., 2019; Wang et al., 2021; Lanza et al., 2022; Yong et al., 2022). However, the potential mechanism associated with the interaction between NaB and gut microbiota-gut-brain communication in PD needs further investigation. In this study, we found that NaB could improve rotenone-induced gut dysfunction, motor deficits, α-synuclein pathology, and dopaminergic neuron loss. Notably, NaB could regulate rotenone-induced gut microbiota dysbiosis and SCFAs metabolism disturbance, and restore the reduction of GLP-1 levels in colon, serum, and substantial nigra.

The gastrointestinal motility disorders in prodromal PD and the occurrence of α-synuclein pathology in gut prior to PD diagnosis indicate that the gut and its connection to the central nervous system plays a potential role in the pathogenesis of PD (Travagli et al., 2020). In this study, we investigated the effect of NaB on gut dysfunction using rotenone-induced PD mice, which has been confirmed to recapitulate the main features of PD-related gut dysfunction (Yang et al., 2017; Perez-Pardo et al., 2019). We observed that the PD mice showed abnormal colon motility and colonic α-synuclein pathology. Indeed, the abnormal colon motility may be associated with the colonic α-synuclein pathology (Fasano et al., 2015). Notably, NaB could protect against rotenone-induced gut dysfunction and ameliorate colonic α-synuclein pathology. Because the influence of NaB on gut function and colonic α-synuclein pathology was rarely reported, our study provided new evidence for the protective role of NaB in the gut dysfunction of PD. Moreover, NaB could improve rotenone-induced motor deficits, α-synuclein pathology in substantial nigra, and protect against the loss of dopaminergic neurons. Similarly, we found that NaB was able to alleviate rotenone-induced expression of α-synuclein *in vitro* in our recent report (Zhang J. et al., 2022). Previous study also reported that NaB treatment could ameliorate motor deficits and α-synuclein pathology in substantial nigra in MPTP-induced PD mice (Hou et al., 2021b). Rotenone and MPTP are commonly used neurotoxins to construct PD mouse model, and rotenone model is more suitable for investigating the α-synuclein pathology in substantial nigra and the role of mitochondrial dysfunction, while MPTP model reproduces the features of PD in terms of dopaminergic neurons loss and neuroinflammation (Zhang J. et al., 2022). Collectively, our study revealed that NaB could improve gut dysfunction and motor deficits, and ameliorate the α-synuclein pathology in colon and substantial nigra in rotenone-induced PD.

The gut microbiota is emerging as a key modulator of neurodegenerative diseases, and a growing body of evidence has linked gut microbiota dysbiosis to the pathogenesis of PD (Nishiwaki et al., 2020). Previous studies reported that regulation of gut microbiota dysbiosis may be a potential therapeutical approach for PD (Sun et al., 2018, 2021; Hou et al., 2021a). In this study, we observed obvious gut microbiota dysbiosis in rotenone-induced mice, which was consistent with previous findings (Yang et al., 2017). NaB treatment could restore the gut microbiota dysbiosis, suggesting that NaB may play a protective role *via* gut microbiota remodeling. In this study, the abundance of Firmicutes was increased, and Bacteroidetes was decreased, and Firmicutes/Bacteroidetes ratio was increased in rotenone-induced mice, which was consistent with previous study (Yang et al., 2017). Higher Firmicutes/Bacteroidetes ratio has been reported to be associated with increased inflammatory conditions (Frank et al., 2007; Jandhyala et al., 2017; Xu et al., 2018), which was associated with the pathogenesis of PD (Marogianni et al., 2020). In this study, NaB treatment restored Firmicutes and Bacteroidetes abundance and Firmicutes/Bacteroidetes ratio. We also found that the abundance of Ruminococcaceae and Lachnospiraceae were decreased in rotenone-induced mice, which were restored with NaB treatment. Similarly, the lower abundance of Ruminococcaceae and Lachnospiraceae has been reported in patients with PD (Romano et al., 2021). Ruminococcaceae and Lachnospiraceae are SCFA-producing bacteria, and generally considered to be beneficial to human health (Brown et al., 2018; Vacca et al., 2020). Thus, NaB may exert beneficial effects by promoting the abundance of SCFA-producing bacteria.

As one of the major metabolites of gut microbiota, SCFAs play a key role in mediating the gut microbiota-gut-brain communication (Dalile et al., 2019). It has been reported that fecal levels of acetic acid, propionic acid, and butyric acid were lower in patients with PD compared with healthy controls, while there were no differences in fecal levels of isobutyric acid, valeric acid, or isovaleric acid (Chen et al., 2022). Yang et al. found that fecal levels of acetic acid, propionic acid, butyric acid, and isobutyric acid were lower in patients with PD, other than valeric acid or isovaleric acid (Yang et al., 2022). In MPTP-induced PD mice, the fecal acetic acid, propionic acid, butyric acid, valeric acid, and total SCFAs levels were lower (Liao et al., 2020). In this study, we found that fecal butyric acid, isobutyric acid, and valeric acid levels were decreased in rotenone-induced PD mice. Previous studies reported that regulation of SCFAs metabolism disturbance could exert protective roles in the pathogenesis of PD (Liao et al., 2020; Hou et al., 2021a). In this study, we demonstrated that treatment with NaB could restore the reduction of fecal butyric acid, isobutyric acid, and valeric acid levels, and increase the levels of acetic acid and propionic acid in rotenone-induced mice. The regulation of NaB on SCFAs metabolism may be partially interpreted by the role of NaB in altering gut microbiota composition, especially increasing the abundance of SCFAs-producing bacteria, such as Ruminococcaceae and Lachnospiraceae.

SCFAs can mediate the gut microbiota-gut-brain communication by modulating secretion of GLP-1 through activation of G protein-coupled receptors (GPCRs) in the enteroendocrine L cells, which are mainly located in distal small intestine and colon (Kaji et al., 2014; Dalile et al., 2019). GLP-1 from the intestine enters circulatory system through intestinal capillaries, and then crosses the blood–brain barrier to enter the brain tissue (Athauda and Foltynie, 2016), and exerts a physiological role by binding to GLP-1R, which is widely expressed in neurons, astrocytes, and microglia (Hölscher, 2014; Kim et al., 2017). In recent years, accumulating evidences suggested that GLP-1 is associated with PD pathogenesis (Hölscher, 2014). Emir et al. reported that the serum GLP-1 levels in patients with PD were lower than those of healthy controls (Emir et al., 2019). In MPTP-induced PD mice, the colonic GLP-1 levels were lower than that in control group (Liu et al., 2017; Sun et al., 2021). In this study, we observed that GLP-1 levels in colon, serum, and substantial nigra were decreased in rotenone-induced PD mice, which was restored with NaB treatment. Similarly, previous study demonstrated that NaB could protect against the reduction of colonic GLP-1 levels in MPTP-induced PD mice (Liu et al., 2017). Because GLP-1 secretion was regulated by SCFAs (Dalile et al., 2019), NaB may restore GLP-1 levels through the regulation of SCFAs metabolism. Several studies have shown that GLP-1 could exert neuroprotective effects in PD by inhibiting neuroinflammation (Fang et al., 2019; Sun et al., 2021). The GLP-1 analog could act on the GLP-1R to exert beneficial effects on mitochondrial function, protein aggregation, neuroinflammation, and synaptic plasticity in multiple experimental models of PD (Athauda and Foltynie, 2016). In this study, the gut microbiota and SCFA metabolism were altered by NaB treatment, and the levels of GLP-1 were increased, suggesting that the GLP-1/GLP-1R pathway might be involved in the communication between NaB and microbiota-gut-brain axis.

## Conclusion

In this study, we found that NaB could ameliorate gut dysfunction, motor deficits, α-synuclein pathology, and dopaminergic neuron loss in PD mice. NaB could regulate gut microbiota dysbiosis and SCFAs metabolism disturbance, and restore the reduction of GLP-1 levels. These findings proved that NaB might exert beneficial effects for the treatment of PD. The potential mechanism might be related to the regulation of gut microbiota dysbiosis. Meanwhile, this study also provided evidence in terms of the complex interactions among gut microbiota, microbial metabolites, and the pathogenesis of PD.

## Data availability statement

The data presented in the study are deposited in the the National Center for Biotechnology Information (NCBI) Sequence Read Archive (SRA), accession number SRP407965.

## Ethics statement

The animal study was reviewed and approved by Research Ethics Committee, Ruijin Hospital, Shanghai Jiao Tong University School of Medicine.

## Author contributions

YZ and SX performed the majority of experiments and wrote the manuscript. YQ, CM, and PA contributed to data analysis. QX and XY designed the study and revised the manuscript. QX provided financial support. All authors contributed to the article and approved the submitted version.

## Funding

This study is supported by the National Natural Science Foundation of China (Grant nos. 81870998, 82171246, 81801254, and 81901283), the Key Field Research and Development Program of Guangdong Province (Grant no. 2018B030337001), the Clinical Research Plan of SHDC (Grant no. SHDC2020CR3012A), and shanghai sailing program (22YF1440200).

## Conflict of interest

The authors declare that the research was conducted in the absence of any commercial or financial relationships that could be construed as a potential conflict of interest.

## Publisher’s note

All claims expressed in this article are solely those of the authors and do not necessarily represent those of their affiliated organizations, or those of the publisher, the editors and the reviewers. Any product that may be evaluated in this article, or claim that may be made by its manufacturer, is not guaranteed or endorsed by the publisher.
